# Notes and Notices

**Published:** 1890-06

**Authors:** 


### NOTES AND NOTICES.

DR. WM. T. BELFIELD, 612 Opera House Building, Chicago, Ill., U. S. A., respectfully solicits information concerning unpublished cases of operations upon the prostate, especially for the relief of the so-called hypertrophy of the organ.

